# Paradigm Shift: Outcomes from a Physician-Scientist Undergraduate Pipeline Program

**DOI:** 10.21203/rs.3.rs-4864555/v1

**Published:** 2024-09-19

**Authors:** Alana C Jones, Tyler R McCaw, Randy L Seay, Brian Sims, Robin G Lorenz

**Affiliations:** University of Alabama at Birmingham Heersink School of Medicine, Department of Medical Education, Physician Scientist Development Office; University of California, Los Angeles; University of Alabama at Birmingham Heersink School of Medicine, Department of Medical Education, Physician Scientist Development Office; University of Alabama at Birmingham Heersink School of Medicine, Department of Medical Education, Physician Scientist Development Office; Genentech, Inc

**Keywords:** pipeline program, physician-scientist workforce, biomedical research, physician-scientistdiversity

## Abstract

Diverse medical and research teams are essential to culturally-responsive care and robust progress of biomedical research. However, structural inequities stymie the entry of trainees from underrepresented in medicine (URiM) backgrounds into the physician-scientist pipeline. The Preparation for Graduate and Medical Education (PARAdiGM) program was designed to provide students from underrepresented backgrounds early exposure to physician-scientist training in the context of ample mentorship and programmatic support. By emphasizing research experience, career exposure, presentation skills, mentorship, and application assistance, PARAdiGM is an incipient experience priming this student group to pursue careers in academic medicine. Since its establishment in 2014, PARAdiGM is already increasing entry of URiM students into the physician-scientist pipeline. Encouragingly, the majority of PARAdiGM alumni have matriculated into US medical schools, of which 16% are currently enrolled in MD-PhD programs. Early outcomes from PARAdiGM suggest that an immersive framework, longitudinal mentoring, and opportunity for self-growth should be incorporated into URiM pipeline programs on a larger scale. In these ways, helping students to envision themselves as members of the physician-scientist community is a step toward breaking down the barriers currently limiting URiM entry into academic medicine.

## Background

Although physician-scientists are uniquely positioned to translate scientific knowledge into improved clinical care, enrollment in physician-scientist training pathways (e.g., MD-PhD programs) has remained largely stagnant.^1^ Further, the diversity of the physician-scientist training pipeline lags behind the physician training overall. In 2022, 15.3% of medical students who matriculated into in US medical schools identified as Black/African American, American Indian/Alaska Native, or Hispanic/Latinx compared to only 11.2% of matriculants to combined MD-PhD programs.^2^ By comparison, these groups comprise ~ 34% of the US general population. Structural inequities resulting in decreased access to support mechanisms, as well as perceived self-limitations, have been shown to contribute to this disparity.^3–5^

To combat these challenges, the University of Alabama at Birmingham Heersink School of Medicine (UABHSOM) established the Preparation for Graduate and Medical Education (PARAdiGM) program in 2014. PARAdiGM introduces undergraduates who are underrepresented in medicine (URiM) and/or have disadvantaged backgrounds to the principles of scientific experimentation, basic research in models of human disease, and the role of physician-scientists in academic medical centers.^6^ A unique feature of PARAdiGM is its longitudinal component: the program takes place over two eight-week summer experiences, along with research presentations and MCAT preparation during the academic year between summers. To date, 46% of PARAdiGM alumni have matriculated into medical school, of which 16% are currently enrolled in an NIH Medical Scientist Training (MD-PhD) Program (MSTP). Among those who did not pursue medicine, 64% obtained graduate degrees and/or are working in biomedical and public health research. Thus, we anticipate that the PARAdiGM model is a potential tool to increase diversity within the physician-scientist and graduate biomedical sciences pipelines.

## Program Enrollment and Structure

**Figure 1:**
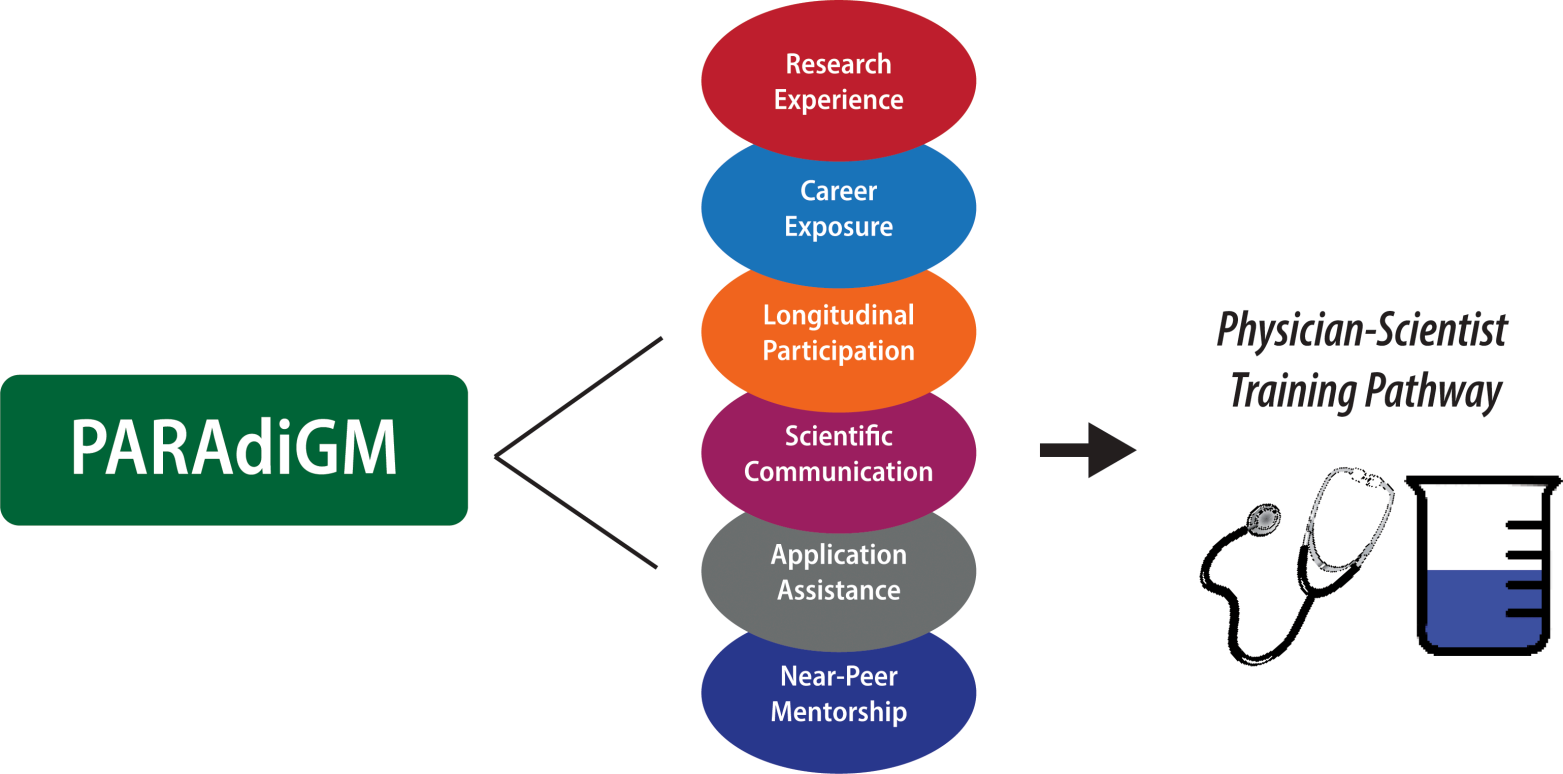
Legend not included with this version.

The tenets of PARAdiGM’s structure are intended to provide students with the requisite experience for a successful acceptance into a physician-scientist training program: (i) research experience, (ii) career exposure, (iii) presentation skills, (iv) near-peer mentoring, and (v) application assistance. These activities provide opportunities to integrate scientific and clinical knowledge in preparation for the physician-scientist path (Fig. 1). Rising undergraduate sophomore, juniors, and seniors are selected from a competitive applicant pool using a holistic review method. We select approximately ten students annually, depending on the number of students who opt to return for a second summer experience. In 2021, however, we doubled our program size in response to the temporary suspension in 2020 caused by the COVID-19 pandemic.

The research experience is the core of PARAdiGM, as it comprises most of the participants’ time. Although the majority of students complete basic research projects, students may also conduct translational and clinical research in alignment with their scientific interests. A particular strength is that the two-summer approach affords students an opportunity to develop lasting mentoring relationships, as they have the option to return to their original lab in the second summer experience. Moreover, students balance their time between to research and weekly shadowing to simulate a day in the life of a physician-scientist. Just as research mentors are selected based on student interests, clinical mentors are ideally in the specialty in which the student plans to pursue and/or is related to the research project.

Additionally, students are provided extensive opportunities to hone their written and verbal communication skills. Students work with their research mentors to prepare a written proposal of the planned project, write an abstract, and submit a final research report in the format of a scientific article. They also attend weekly workshops on various elements of scientific writing and can receive feedback on their drafts from the writing instructor who leads these workshops. Furthermore, students sharpen their oral presentation skills through weekly career development workshops, including one in which each student is required to give a “chalk talk” or “three-minute thesis” — a summary of their research project in front of their peers—and receive feedback from program leadership. Other sessions include “How to Create a Research Poster” and an informal practice session in preparation for a university-wide undergraduate summer research symposium. These experiences serve as primers for PARAdiGM students’ presentations at the Annual Biomedical Research Conference for Minoritized Scientists (ABRCMS). Participation in this national conference not only offers students additional opportunities for research presentation and networking, but it also provides a sense of accomplishment, thus reaffirming that they can become physician-scientists.

In addition to having faculty-level clinical and research mentors, students are paired with a current MD-PhD trainee. The mentor-mentee pairs meet weekly to discuss goals, provide feedback on abstract and poster drafts, and generally be a source of support for the student. In addition to individual mentors, designated MD-PhD trainees also facilitate formal application assistance through a “Preparation for Medical School” course. The curriculum includes professionalism, study skills, strategies for navigating medical school interviews, and high-yield content review for the Medical College Admissions Test (MCAT) in a flipped classroom model. These designated mentors also proctor an official practice MCAT and facilitate the distribution of a commercially available set of MCAT review books, both provided at no cost to students. Second-year students also provide feedback on the curriculum each year and have the opportunity co-teach some sessions as well, creating a feed-forward loop of mentoring.

## Program Evaluation and Outcomes

We evaluated demographic characteristics and post-participation outcomes through 2021, as these individuals have all completed their undergraduate training. These outcomes are tracked via updates to a shared LinkedIn group for PARAdiGM alumni, as well as informally through near-peer mentors. Moreover, we also collected subjective feedback from program participants through the duration of their participation. From 2014 to 2019, students completed pre- and post-participation surveys to evaluate interval change in their interest in a physician-scientist career. These surveys were collected via Qualtrics, an online platform that tracks responses while maintaining anonymity of results and verifying the quality of response data. The program was temporarily halted in 2020 due to the COVID-19 pandemic. And in 2021, we discontinued our internal survey in favor of the Student Assessment of their Learning Gains (SALG) instrument.^7^ In both surveys, responses are ranked on a Likert scale of 1 to 5, with 1 being least favorable/ “no gains” and 5 being most favorable/ “great gain”. We report the mean and standard deviation for each response category.

Demographic characteristics are presented in Table 1. Of 60 total participants from 2014 to 2021, 62% identified as women. Approximately 67% of students were African American or Black, 27% as Hispanic or Latine, and 5% Native American/Alaska Native. About 68% of participants reported a disadvantaged background. More than half of the students attended Historically Black Colleges and Universities (HBCUs) and/or Minority Serving Institutions (MSIs), and matriculant GPAs ranged from 3.1 to 4.0. Although 93% of students majored in science, technology, engineering, or mathematics (STEM) or health-related fields, approximately 7% obtained undergraduate degrees in humanities and/or social sciences. Furthermore, prior to 2020, more than 70% of eligible individuals opted to return for a second summer experience. However, given a lower rate among the 2021 cohort (5%), the overall rate of return was 47%.

Participant outcomes are summarized in Table 2. Most PARAdiGM alumni (58%) are pursuing further training. Four individuals were lost to follow-up, and their status is unknown. However, three alumni are currently enrolled in MD-PhD (MSTP) programs, of which all have obtained NIH F30/F31 predoctoral fellowship awards. Approximately 38% of students matriculated into MD or DO programs, and approximately half of these individuals are currently enrolled in residency training programs. Among those who did not pursue medicine, one-third are enrolled in biomedical science or public health postgraduate programs. And among those who have entered the workforce, 60% have careers in biomedical or clinical research, biotechnology, pharmaceutical sciences.

Moreover, survey responses reflected increased comfort in research presentation and a greater understanding of academic medicine (Table 3). Students also indicated that PARAdiGM helped them decide what to pursue after college. Additionally, students reported that the program furthered their desire for a career in research, with an increasing trend over time. In the SALG survey (post-2019), students consistently reported “good” to “great” gains in the following categories: thinking and working like a scientist; personal gains in confidence to do research; and acquisition of technical skills related to research, such as written and oral communication, literature review, and time management. Notably, all respondents reported “great gain” in feeling like a scientist (data not shown).

## Conclusions

Early outcomes suggest the longitudinal and immersive nature of PARAdiGM, continuous contact with mentoring teams, and opportunity for self-growth are important components that should be adapted to URiM pipeline programs on a larger scale. Effective mentoring relationships are particularly critical for these students given the distinct set of challenges faced by URiMs across the physician-scientist pipeline.^5^ To this end, PARAdiGM provides a wealth of support, including near-peer mentors who can alleviate anxieties rooted in starting a new training path. Although relatively new, PARAdiGM outcomes are auspicious, with the majority of alumni currently enrolled in physician-scientist and physician training programs. This percentage is comparable to a more established pipeline program, the Gateways to the Laboratory, where 17% of program participants enrolled in a combined MD-PhD degree program.^8^ And to date, PARAdiGM alumni boast 137 peer-reviewed publications and dozens of local and national presentation awards, underscoring the value of the PARAdiGM experience, as well as trainee confidence in their ability to make meaningful, scientific contributions.

Structural inequities are the primary barrier to URiM entry into the physician-scientist pipeline, and their psychological impacts (e.g., stereotype threat) may exacerbate these disparities. ^9,10^ Previous studies have demonstrated that near-peer mentoring affords a sense of social support and increases self-efficacy and motivation among UriM students aspiring to enter medical post-secondary education. ^11^ Moreover, socioeconomic inequalities and financial hardship contribute to disparities in MCAT performance. ^4^ As PARAdiGM students come from disproportionately disadvantaged backgrounds, we seek to alleviate this financial burden by providing a set of commercial MCAT review books—approximately $300 per set—and a practice MCAT exam at no cost to students, in addition to housing and a summer stipend. This investment not only removes a material barrier to a career in medicine, but also boosts URiM trainees’ confidence in their ability to succeed.

Furthermore, students’ perceived self-efficacy in skills fundamental to academic medicine undoubtedly influences their decision to enter the pipeline. PARAdiGM therefore provides a framework to build confidence in these areas, as students are first taught techniques for scientific communication, then provided opportunities to practice, and later able to compete for awards at internal and national conferences. Success of this approach was recounted by one student: “this program has become the driving force of my new direction into the graduate school realm, that I had not previously thought was possible”. Importantly, as PARAdiGM trainees become more advanced in their training, they can be a source of support for more junior trainees, creating a feed-forward loop of mentoring. One student noted that participation in PARAdiGM allowed them to embrace the “ability to be a role model for other kids like me who do not think they can do it”. At the same time, they often establish long-term relationships with their own near-peer mentors, as these relationships further normalize physician-scientist training and help them envision themselves in the same position.

Overall, initial outcomes are promising although there are some limitations. As a single-site program, it is unclear how well these interventions would transfer to larger summer programs or those that are not affiliated with physician-scientist training programs (e.g., MSTP). Additionally, trainees in a post-pandemic environment are likely to have different programmatic needs that are not addressed by our current structure, in considering differences in outcomes from the 2021 cohort in comparison to previous years. Not only were these individuals more likely than previous cohorts to pursue clinical or biomedical research postgraduate training, but they were also less likely to return for a second summer experience. It is possible, though, that participation in PARAdiGM provided these students with a representative experience of being a physician-scientist, and they ultimately chose to pursue a different course. If that it is the case, participation in PARAdiGM was still a positive experience for these students’ identity and career formation. Furthermore, a strength of the PARAdiGM structure is its ample commitment to small-group and individual training, as it allows trainees to form close bonds and support each other through the training pipeline. In the 2021 cohort, however, this effect may have been diluted, and as such, the PARAdiGM approach may not be generalizable to larger programs. Finally, the long-term impacts of these interventions are not yet known given the relative infancy of the program. Still, it is likely that PARAdiGM will continue to pave a new avenue for URiM students to enter the physician-scientist and biomedical research pipelines.

In this study, we highlight PARAdiGM as a multi-pronged, interdisciplinary model for approaching the “leaky pipeline” of physician-scientist diversity. By offering research experience, ample access to mentorship, formal instruction in scientific communication, and application assistance through a longitudinal format, this program has produced dozens of physicians and physician-scientists. Implementation on a larger scale is likely to reduce barriers to entry for URiM trainees and increase physician-scientist diversity.

## Figures and Tables

**Table 1:** Baseline characteristics of PARAdiGM participants, 2014–2021

Characteristic Mean(SD)/N(%)	Total (N = 60)
Age	20(1)
Gender, Women	37(62)
Race	
AA/Black	40(67)
AI/AN	3(5)
Other	5(8)
White	12(20)
Hispanic/Latine	16(27)
Disadvantaged	41(68)
Enrolled at Minority Serving Institution	31(52)
STEM or Health-Related Major	56(93)
Returned for Second Summer Experience	15(47)

**Table 2:** Postgraduate outcomes of PARAdiGM participants, 2014–2021

Year	2014	2015	2016	2017	2018	2019	2021	Total
Total	5	8	8	8	5	7	19	60
Further Training	3(60)	8(100)	5(63)	5(63)	3(60)	4(57)	7(37)	35(58)
Physician-Scientist	2(67)	1(13)	0(0)	0(0)	0(0)	0(0)	0(0)	3(9)
Clinical-Medicine	0(0)	7(87)	3(50)	3(60)	2(67)	2(50)	3(43)	20(57)
Clinical-Other	0(0)	0(0)	0(0)	0(0)	0(0)	1(25)	1(14)	2(6)
Biomedical Sciences	1(33)	0(0)	3(50)	2(40)	1(33)	1(25)	1(14)	9(26)
Public Health	0(0)	0(0)	1(13)	0(0)	0(0)	0(0)	2(29)	3(9)
Industry	1(20)	0(0)	1(13)	1(13)	2(40)	1(14)	2(11)	8(13)
Research	2(40)	0(0)	1(13)	1(13)	0(0)	0(0)	3(16)	7(12)
Other	0(0)	0(0)	1(13)	1(13)	1(20)	2(29)	7(37)	12(20)

N(%). For sub-categories, percentages are based on the total within that category alone, not the total of all participants. Students who participated in 2 summers were only tabulated by the year of their first summer.

**Table 3:** Student interest in a physician-scientist career path, 2014–2019

Category	Pre-Participation	Post-Participation	% Change
Clinical medicine	4.8(0.5)	4.9(0.4)	+ 2.1
Basic science research	3.6(0.8)	3.4(1.0)	−7.1
Clinical research	4.3(0.7)	4.3(0.7)	−0.2
Translational research	3.6(1.0)	3.7(0.9)	+ 2.3
Any research	3.3(1.3)	3.8(0.9)	+ 16.8
Teaching	3.1(1.1)	3.7(1.2)	+ 21.2
Leadership	4.1(0.8)	4.7(0.5)	+ 13.7
Mentoring	4.4(0.7)	4.6(0.5)	+ 4.7
Career at large academic medical center	4.2(0.7)	4.4(0.7)	+ 5.3
Private practice	3.9(0.9)	3.5(0.9)	−9.9

Aggregate survey results presented as mean(standard deviation) of responses. Pre: pre-participation survey responses. Post: post-participation survey responses. Likert scale ranked 1–5 with 1 indicating “not interested at all” and 5 indicating “very interested”
